# The present and the future of benzydamine: expert opinion paper

**DOI:** 10.3389/froh.2023.1191319

**Published:** 2023-06-16

**Authors:** Paolo Bossi, Cristina Gurizzan, Orlando Guntinas-Lichius, Razvan Hainarosie, Davide Lancini

**Affiliations:** ^1^ Medical Oncology, Department of Medical and Surgical Specialties, Radiological Sciences, Public Health, University of Brescia, Brescia, Italy; ^2^ Department of Otorhinolaryngology, Jena University Hospital, Jena, Germany; ^3^ Department of Otorhinolaryngology, “Carol Davila” University of Medicine and Pharmacy, Bucharest, Romania; ^4^ Unit of Otorhinolaryngology-Head and Neck Surgery, ASST Spedali Civili of Brescia, Brescia, Italy

**Keywords:** benzydamine, oral lichen, burning mouth disorder, sore throat, mucositis and stomatitis

## Abstract

**Objectives:**

Benzydamine is a compound indicated in the treatment of symptoms of irritation/inflammation of the oropharyngeal cavity, including those associated with pain. Objectives of this expert opinion narrative review is to summarize current indicated benzydamine applications and elicit further ones to be explored.

**Materials and methods:**

In this expert opinion paper, evidences underlying the mechanism of action and clinical application of benzydamine are reviewed. Insights are also provided on possible new clinical applications of the drug and new formulations.

**Results:**

Current recognized indications of benzydamine include: relief of symptoms associated with inflammatory conditions of the mouth and oropharynx, symptomatic treatment of gingivitis and stomatitis, oral mucositis induced by chemotherapy and/or radiotherapy and post operative sore throat. In addition, among new applications that need to be explored experts embed: oral lichen planus, burning mouth syndrome, post intubation sore throat, antifungal action and new anticancer target agents induced mucositis.

**Conclusions:**

Benzydamine is a very versatile compound able to play an auxiliary and adjuvant role in the prevention and treatment of oral cavity/oropharynx disorders. According to experts’ opinion there is the need to design clinical trials to highlight novel possible applications of benzydamine and implemented translational analyses to fine-tune patients’ selection and open future research scenarios.

## State of the art: use in prevention and treatment settings

Benzydamine is an indazole derivative, non-steroidal agent with local anaesthetic and analgesic properties. Consistent and reliable pre-clinical and clinical data demonstrate both its safety and efficacy (1, 2).

Nowadays, the main indication for benzydamine use is the relief of symptoms associated with inflammatory conditions in the mouth and oropharynx (3). According to the clinical indications in the various countries where benzydamine is marketed it is also indicated for the symptomatic treatment of gingivitis and stomatitis, oral mucositis (OM), induced by chemotherapy and/or radiotherapy, in patients with head and neck (HN) cancer, as well as for post operative sore throat after oropharyngeal-laryngeal surgery, including endotracheal intubation for any kind of surgery (4).

The anti-inflammatory effect of benzydamine is mainly due to its inhibitory activity on the production of proinflammatory cytokines such as TNF-α, IL-1β, MCP-1. Two studies by Sironi (5, 6) demonstrated how benzydamine is able both *in vitro* and *in vivo* to inhibit inflammatory cytokines production in mononuclear phagocytes (PBMC) exposed to different triggers. In particular, benzydamine is effective in reducing the production of proinflammatory cytokines, without affecting anti-inflammatory factors (reduction of TNF-α and IL-1β, with no impact on IL-6 and IL-8 levels). Together with reduction of inflammatory cytokines, benzydamine also exerts modulation of the inflammatory status through different actions: reduction of vascular permeability caused by the release of histamine, acetylcholine, serotonin and epinephrine; inhibition of platelets aggregation and thrombus formation; inhibition of human polymorphonuclear leukocytes degranulation; inhibition of human monocytes migration (1).

In addition to this complex modulation of the inflammatory status, there is another less explored but interesting action of benzydamine. It has been reported to attenuate nociceptor excitability and local transmission of painful stimulus through a block of sodium channels (7). During the recent Biophysical Society Annual Meeting 2022, Ferrer-Montiel and colleagues reported how benzydamine exerts pain relief not only by reducing inflammation cascade, but also by reducing the inflammatory mediated neuronal signalling, closing the local circle of inflammation and pain (8).

Moreover, benzydamine demonstrated *in vitro* anti-microbial activity. Fanaki et al. (9) showed how benzydamine has a non-specific antibacterial activity and most important it is active against strains resistant to broad-spectrum antibiotics. Also, when combined to tetracyclines and chloramphenicol benzydamine exerts a synergistic antibacterial effect by increasing the antibiotic bacterial uptake (10).

Several clinical trials showed that topical benzydamine prevents postoperative sore throat (POST). In a meta-analysis of thirteen randomized controlled trials involving 1,842 patients, benzydamine was associated with a lower incidence of postoperative sore throat than patients who did not receive analgesia. It was also associated with a reduced incidence of POST when compared with lidocaine. Its good safety profile was confirmed (11).

Moreover, during the recent National Congress of the Italian Association for the study of pain preliminary results of the recently completed phase IV BePaiR study (NCT04941976) were presented. This study was designed to assess the efficacy of two different formulations of benzydamine hydrochloride (0.3% oromucosal spray or 3 mg lozenges) on non-complicated sore throat pain relief, at 2 min after a single dose administration. Benzydamine 0.3% spray proved to be non-inferior in reducing sore throat pain with respect to benzydamine 3 mg lozenges, starting already from 2 min after a single administration and lasting up to up to 4 h. The clinical efficacy after 7 days of treatment was also demonstrated (12).

Finally, benzydamine exerts a role in the prevention and treatment of chemoradiotherapy-induced OM in HN cancer patients (13). According to the recent published MASCC guidelines for the management of cancer therapy-induced mucositis, benzydamine mouthwash is recommended for the prevention of OM when administering a moderate dose RT (<50 Gy). It is also suggested in case of concomitant chemo-radiotherapy (14).

Table 1 summarize the main indications across different countries for different benzydamine formulations in sore throat and other inflammatory conditions of the oral cavity.

**Table 1:** Indications for different benzydamine formulations in sore throat and other inflammatory conditions of the oral cavity.

Brand name	Active substance	Pharmaceutical form	Clinical indication	Posology^a^
TANTUM VERDE spray 0.3%	Benzydamine chlorohydrate	Spray solution for oral mucosa	It is indicated for pain relief and irritation of throat and mouth It is indicated for symptomatic treatment (pain, irritation) in post-operative conditions, after surgical procedures in otolaryngology, after intubation^b^	Adults: 2–4 nebulizations, 2–6 times a day 1 puff is equivalent to 0.510 mg of benzydamine hydrochloride; thus, the first dose will be equivalent to 1.02 mg and total daily dose is ranging from 2.04 to 12.24 mg benzydamine hydrochloride maximum in adults
TANTUM VERDE spray 0.15%	Benzydamine chlorohydrate	Spray solution for oral mucosa	It is indicated for pain relief and irritation of throat and mouth	Adults: 4–8 nebulizations, 2–6 times a day Children (6–12 years): 4 nebulizations, 2–6 times a day Children (under 6 years): 1 nebulization far each 4 kg of body weight, up to a maximum of 4 nebulizations, 2–6 times a day 1 puff is equivalent to 0.255 mg of benzydamine hydrochloride, thus the first dose will be equivalent to 1.02 mg and total daily dose is ranging from 2.04 to 6.12 mg benzydamine hydrochloride maximum in children and to 12.24 in adults
TANTUM VERDE mouthwash	Benzydamine chlorohydrate	Mouthwash (1.5 mg/ml oral solution)	It is indicated for pain relief and irritation of throat and mouth It is indicated for symptomatic treatment of oromucosal inflammation including secondary conditions associated with radiotherapy (RT) such as mucositis^b^	15 ml 3 times a day
TANTUM VERDE Lozenges 3 mg (different flavors)	Benzydamine chlorohydrate	Lozenge 3 mg	It is indicated for pain relief and irritation of throat and mouth	Adults and children over 6 years of age: 1 lozenge three times a day

^a^ Please note that the POSOLOGY reported in the table is based on the benzydamine ltalian SmPCs (Summary of Product Characteristics); please refer to the SmPC of each Country to check the relevant posology.

^b^ These indications are present only in the SmPC of some Countries; please refer to the local SmPC.

## New potentials in the benzydamine use

### Oral lichen planus

Oral lichen planus (OLP) is a chronic inflammatory disease of the mucosa of the oral cavity based on T-cell mediated autoimmune process. The estimated prevalence of OLP in the general population is 3.6% and the spectrum of disease presentation includes the following types: papular, reticular, atrophic, blistering, erosive and plaque. Being a chronic disease, the management of OPL is mainly related to the elimination of precipitating or provoking factors and relief of symptoms (15). Topical steroids remain the first-line treatment for OLP, while systemic steroids are reserved for acute exacerbation and in case of unresponsiveness to topical formulations. This class of drugs exerts its anti-inflammatory action by causing a reduction in pro-inflammatory cytokines and immune cells at the site of inflammation (16). However, these drugs are not devoid of adverse events (AEs). The most common is the occurrence of oral candidiasis, observed in about 25%–55% of patients. Other recognized local adverse effects include burning mouth, hypogeusia, oral hairy leucoplakia and hypersensitivity reactions to the drug. Systemic adverse effects are rare, but not unavoidable. These side events are related to systemic absorption of the drug and the consequent inhibition of the hypothalamic-pituitary-adrenal axis and secondary adrenal insufficiency (17).

In this scenario the use of benzydamine for OLP may represent an interesting new strategy to be employed in a multimodal approach to reduce symptoms. Given its role in reducing anti-inflammatory cytokines production and modulating the inflammatory status, different formulations of benzydamine may play an adjuvant role in the management of OPL. In addition, reducing the local transmission of painful stimuli may help relieve pain due to oral lesions. Moreover, thanks to its anti-microbial and anti-fungal properties benzydamine may reduce the burden of oral candidiasis caused by topical oral corticosteroids. Therefore, the design of new clinical trials aimed at evaluating the auxiliary and adjuvant role of benzydamine in the treatment of OPL could be very interesting.

### Burning mouth syndrome

Burning mouth syndrome (BMS) is defined as an intraoral burning or dysesthetic sensation, recurring for more than 2 h per day and for more than 3 months, without evident causative lesions during the clinical examination. Although the etiology remains unknown, there are increasing evidences underlying a neuropathic genesis of pain in BMS caused by peripheral and central nervous system changes (18). So far, no consensus has been achieved about the best treatment of BMS. Most commonly used therapeutic approach includes tricyclic antidepressants, α-lipoic acid, clonazepam, and cognitive-behavioural therapy (19). According to a network metanalysis (20) aimed at evaluating as primary outcome pain relief or burning sensation, only clonazepam is likely to reduce the pain of BMS when compared with placebo.

The efficacy of benzydamine for oral pain relief was evaluated in a randomized controlled trial involving patients with idiopathic BMS. Thirty patients were randomly assigned to one of 3 management modalities: oral rinse solution of benzydamine 0.15% 3 times a day for 4 weeks, placebo 3 times a day for 4 weeks or any kind of treatment. The study showed no significant differences between the groups (21).

Nevertheless, there is still a strong rationale for further evaluating the use of benzydamine in this setting. Benzydamine is known to block Na+ channels expressed in sensory neurons, and therefore attenuate nociceptor excitability by preventing the generation and propagation of action potentials through block of Na+ channels (7). In the light of these data and the neuropathic genesis of pain observed in BMS, new trials aimed at evaluating the efficacy of benzydamine as a potential additional weapon in relief pain of BMS patients could provide new clinical evidence on clinical indications other than inflammatory conditions of the throat and the mouth.

### Post intubation sore throat

Postoperative sore throat (POST) represents one of the most common side effects after endotracheal intubation for any surgical reason. The estimated overall incidence ranges from 22% to 62% in the adult population, while from 24% to 44% in the pediatric population. The peak incidence of POST is reported between 2 and 4 h after extubation (22). POST is induced by direct mucosal inflammation caused by the mechanical trauma of endotracheal intubation. Recognized risk factors of POST include the presence of upper respiratory tract infection, duration of anaesthesia, intubation without neuromuscular blockers, number of intubation attempts, high cuff pressure, and the anaesthesiologist's experience (22).

The role of benzydamine in this setting has been largely explored. In 1987 Mazzarella et al. (23) assessed the efficacy of benzydamine 0.3% spray over placebo in relief POST when applied just before intubation and then every 3 h for 3 days following surgical procedure. Interestingly, together with clinical assessment of objective signs and subjective reported symptoms, also cytological differences confirmed these results. Two cytological samples were obtained from each patient by brushing the vocal cord, before intubation and after extubation, and at microscopical evaluation patients receiving benzydamine presented with a statistically significant less inflamed cytology (i.e., less inflammatory cells, blood cells and foamy cells).

Chang et al. (24) demonstrated the prophylactic role of benzydamine spray on POST after intubation with a double-lumen endobronchial tube. Applied on oropharyngeal cavity before intubation, benzydamine compared with placebo reduced the onset (at 1, 6 and 24 h after surgery) and the intensity of pain according to visual analogue scale (VAS). The role of benzydamine in reducing the incidence and severity of sore throat 24 h after surgery/extubation was confirmed in a systematic review and meta-analysis of 13 randomized controlled trials involving 1,842 patients (11). On the contrary, the same purpose needs to be further explored in pediatric population (25).

Even if POST has been largely evaluated, we believe that some issues still need to be addressed. First, in case of prophylactic POST treatment benzydamine is just sprayed on endotracheal tube cuff; however, it is well known how the source of pain after intubation is represented by traumatic inflammation of the laryngeal subsites that are in direct contact with the endotracheal tube, i.e., epiglottis, posterior commissure and vocal cords. In this regard, application of the drug through laryngoscopy directly to the target areas could provide additional benefit, even considering the availability of a new drug formulation such as gel. Moreover, thinking about tonsillectomy as an extremely painful procedure and at risk of postoperative bleeding, the development of new compounds able to reduce inflammation and bleeding without gargles is really intriguing. The potential auxiliary role of a novel combination of benzydamine with fibrin glue, could be of great interest in the multimodal approach to these unmet needs.

### Antifungal action

A recent work by Ardizzoni et al. (26) demonstrated *in vitro* the ability of benzydamine mouthwashes to impair *Candida Albicans* adhesion, biofilm formation, and biofilm regrowth and persistence. Moreover, as interestingly stated by the authors counteract *Candida* colonization may favour oral cavity health by reducing the growth and persistence of oral pathogens such as *Streptococcus mutans*.

Even if not confirmed by *in vivo* studies, these data highlight the possibility of using benzydamine to prevent oral candidiasis, as well as to treat biofilm-related *Candida* infections. This application may be of particular interest in frail, elderly, and immunocompromised patients. Therefore, clinical trials to address the need of reduce oral candidiasis in the aforementioned population could potentially expand the scope of benzydamine as an adjuvant antifungal tool.

### Targeted agents (mTOR inhibitor) and antibody drug conjugates (ADC) induced mucositis

In recent years, substantial progress in the unveiling of mechanisms underlying oncogenesis has led to a significant improvement in the anti-cancer therapy scenario.

Oral toxicities of targeted therapies are not unusual in clinical practice and present peculiar features which differ from those observed with chemotherapy (27). In this setting, the best described oral toxicity is that caused by mammalian target of rapamycin inhibitors (mTORi: everolimus, temsirolimus, deferolimus) (28). Mucositis induced by mTORi represents the most prevalent and dose-limiting adverse event, with an overall incidence of any grade according to Common Terminology Criteria for Adverse Events (CTCAE) of up to 52.9% (27). Mucositis can negatively impair adherence to the cancer treatment and affect the patient's quality of life, aspects that configure it as a non-negligible toxicity. Therefore, early management and prevention are crucial and include good oral hygiene and prophylactic use of alcohol-free dexamethasone mouthwash (29).

Another novel therapeutic breakthrough is represented by antibody drug conjugates (ADCs), which combine the potent cytotoxicity of chemotherapy with the antigen-specific targeted approach of antibodies into one single molecule. For instance, in the two studies BEGONIA (NCT03742102) and Tropion PanTumor01 (NCT03401385) evaluating the ADC datopotamab-deruxtecan in advanced triple negative breast cancer, grade 3 stomatitis has been reported in 14% and 9.1% respectively.

Considering these data and taking in account that benzydamine was able to relieve symptoms of OM induced by radio-chemotherapy, it would be of great interest the possibility of studying its efficacy in patients who are treated with mTORi and ADCs.

## Defining ideal benzydamine trials endpoints and how to measure them

The most crucial point when designing a clinical trial on pain is for sure to define its endpoints and how to measure them. In this regard, new possible benzydamine trials need a fine tuning in order to better answer to unsolved questions.

Among the most adopted endpoints to evaluate the efficacy of different benzydamine formulations intended as pain relief are frequency of pain, its duration and severity. If frequency and duration of pain can be measured in a reliable and reproducible manner, the same is not true for severity. In fact, pain is typically assessed with scales including Numerical Rating Scale (NRS), Visual Analogue Scale (VAS), Verbal Rating Scale (VRS), and Faces Pain Scale-Revised (FPS-R); however, ratings on these measures may be influenced by non-pain intensity factors (30). Thong et al. (31) evaluated the associations among pain intensity scales and non-pain intensity factors (depressive symptoms, pain unpleasantness, catastrophizing, and interference). They showed how VAS and NRS appear to be most similar and less influenced by non-pain intensity factors than VRS or FPS-R. Given these considerations, pain relief as an efficacy endpoint needs to be accurately measured in order to obtain reliable and consistent results for the use of benzydamine.

Other underestimated endpoints should be considered when evaluating benzydamine. For instance, reducing the consumption of analgesic and anti-inflammatory drugs in favour of benzydamine compound could decrease the rate of adverse events related to their use.

In addition, it would be interesting to indirectly assess the efficacy of benzydamine compared with different compounds. For example, the reduction of indirect costs can be quantified to delineate the whole cost-effectiveness profile of the treatment.

In this scenario the use of new tool such as patient-reported outcomes measures (PROMs) to better describe patient experience, increase research robustness, maximize economic value and improve patient outcomes is conceivable and needs to be systematically implemented in the design of new benzydamine trials (32, 33). One of the most helpful tool could be the Patient-Reported Outcome Common Terminology Criteria for Adverse Events (PRO-CTCAE), which can detect adverse events that other biomedical measures may not (34).

At the time of writing this paper, according to ClinicalTrials.Gov only one study aiming at evaluating benzydamine gargles on severity and duration of post-operative sore throat was actively recruiting (NCT05343429). Thus, enhancing the need of designing new trials on the possible applications of benzydamine in clinical practice.

A phase IV international clinical study on Benzydamine hydrochloride in pediatric population (6–12 years) will be performed in Poland, Hungary and Romania and has been already approved by the Competent authorities (EudraCT number: 2022-003285-20; Sponsor Angelini). The purpose of this study is to update clinical data and available information on the efficacy and safety of two different formulations of benzydamine hydrochloride (0.15% spray and 3 mg lozenges) in pediatric patients with sore throat.

Another phase IV international clinical study is planned to understand the role of benzydamine in the treatment of postoperative sore throat in adults undergoing general anesthesia.

## Conclusion and new opportunities

So far, benzydamine has proven to be a very versatile compound. Different formulations can play a role as adjuvant and auxiliary weapon in various settings for the prevention and treatment of oral cavity/oropharynx disorders. However, we believe there are further interesting possibilities to be explored.

First, new gel drug formulations may be evaluated. For instance, the gel formulation could allow precise application of the drug on small surfaces, such as small aphthous lesions of the oral cavity or specific pharyngeal subsite before and after intubation. In addition, the gel formulation could allow the drug to remain in contact with the affected area for a longer time, favouring its effectiveness.

It would also be of great interest to study possible combinations with benzydamine, for example, with multivitamin complex or alpha-lipoic acid in case of BMS.

Finally, there's an increasing need to pursue with translational research to unveil new potential biomarkers and predictors. In case of benzydamine, its role in reversing the proinflammatory balance by reducing some cytokines (i.e., TNF-α and IL-1β) in favour of others (i.e., IL-8) may be studied also in saliva. Therefore, salivary cytokines levels analyses should be considered in different trials.

In conclusion, experts agree in considering benzydamine an intriguing compound that should be considered within a multimodal approach to address the symptomatology of different oral cavity/oropharynx disorders. The design of new studies to further explore its application is needed.

## Data Availability

The original contributions presented in the study are included in the article, further inquiries can be directed to the corresponding author.
